# Modification of Kong’s Lateral Canthotomy Simulation Trainer for Added Realism and Tonometry Practice

**DOI:** 10.7759/cureus.106595

**Published:** 2026-04-07

**Authors:** Stephanie Torrez, Hana-Joy E Hanks, Lizveth Fierro, Alana E Harp, Timothy P Young

**Affiliations:** 1 Emergency Medicine, Loma Linda University Medical Center, Loma Linda, USA; 2 Emergency Medicine, Loma Linda University School of Medicine, Loma Linda, USA; 3 Pediatric Emergency Medicine, Loma Linda University Medical Center, Loma Linda, USA

**Keywords:** cantholysis, intraocular pressure, lateral canthotomy, medical education, simulation, tonometry

## Abstract

Lateral canthotomy with cantholysis is an emergency procedure that relieves critically elevated intraocular pressure. This procedure is uncommon, so opportunities to observe or practice it on a live patient are limited. There is a spectrum of accessible, inexpensive simulation models available for practicing the procedure. One innovative trainer uses a table tennis ball as the globe, a rubber band as the canthal tendon, and foam tape as skin. We modified this model by replacing the table tennis ball with an endotracheal tube cuff. This allowed learners to practice measuring intraocular pressure with a handheld applanation tonometer as an independent procedure and as part of performing a lateral canthotomy. The cuff provided a realistic tactile feel. The cuff may rupture if handled improperly, reinforcing the importance of careful technique. Successful procedural completion is signaled by a measurable decrease in intraocular pressure. We share our modifications in this article and describe users' experience with the trainer as reported in a survey. The trainer improved learners’ comfort with both tonometry and lateral canthotomy with cantholysis. We used our trainer in a teaching session with 53 participants who completed a pre- and post-session Likert scale survey. Median comfort level for performing tonometry before the session improved from 4/5 (somewhat comfortable; IQR: 3-5) to 4.5/5 (somewhat comfortable/very comfortable; IQR: 4-5, P = 0.002) after the session. Median comfort level for performing lateral canthotomy with cantholysis before the session improved from 2/5 (somewhat uncomfortable; IQR: 1-3) to 4/5 (somewhat comfortable; IQR: 3-4, P < 0.00001) after the session. Our modifications to the trainer increased the cost from 5 US$ to 15 US$. Emergency medicine and ophthalmology educators may find our modified model to be a helpful teaching tool for this rare procedure.

## Introduction

Lateral canthotomy with cantholysis is a rarely performed, vision-saving procedure that must be executed in a timely manner when a patient develops orbital compartment syndrome (OCS). This condition occurs when a patient’s intraocular pressure (IOP) rapidly rises in the confined space of the eye socket, compressing the optic nerve and resulting in decreased optic nerve perfusion. IOP can rise as a result of excessive fluid, mass, or even air. Most commonly, OCS develops as a result of hemorrhage in the setting of facial trauma, but it can also occur as a result of ocular surgery/injections, metastases or tumors, infection, and third spacing in the setting of burns [1]. While the normal range of eye pressure is less than 20 mmHg, a measurement of 40 mmHg has been suggested as a threshold for procedural intervention, while also considering clinical indicators, such as vision loss and afferent pupillary defect [2]. IOP can be measured before and after the procedure to confirm effective release of the canthal tendon, which ultimately serves to provide additional space for a disease process to occur without causing further damage to the optic nerve.

Training learners to become procedurally competent with such a rarely encountered skill can be challenging for medical educators. In the past, we struggled to provide opportunities for tonometry and lateral canthotomy practice in our training program. Simulation is an established modality for teaching rare procedures [3]. Kong and colleagues described a cost-effective lateral canthotomy trainer that is built from accessible items [4]. We have used this model successfully for several years, but found that the rigid globe lacked realism, and the model did not allow us to measure IOP. We modified Kong's trainer by using the cuff of an endotracheal tube (ETT) to simulate the globe. We describe the modification below.

## Technical report

**Figure 1:**
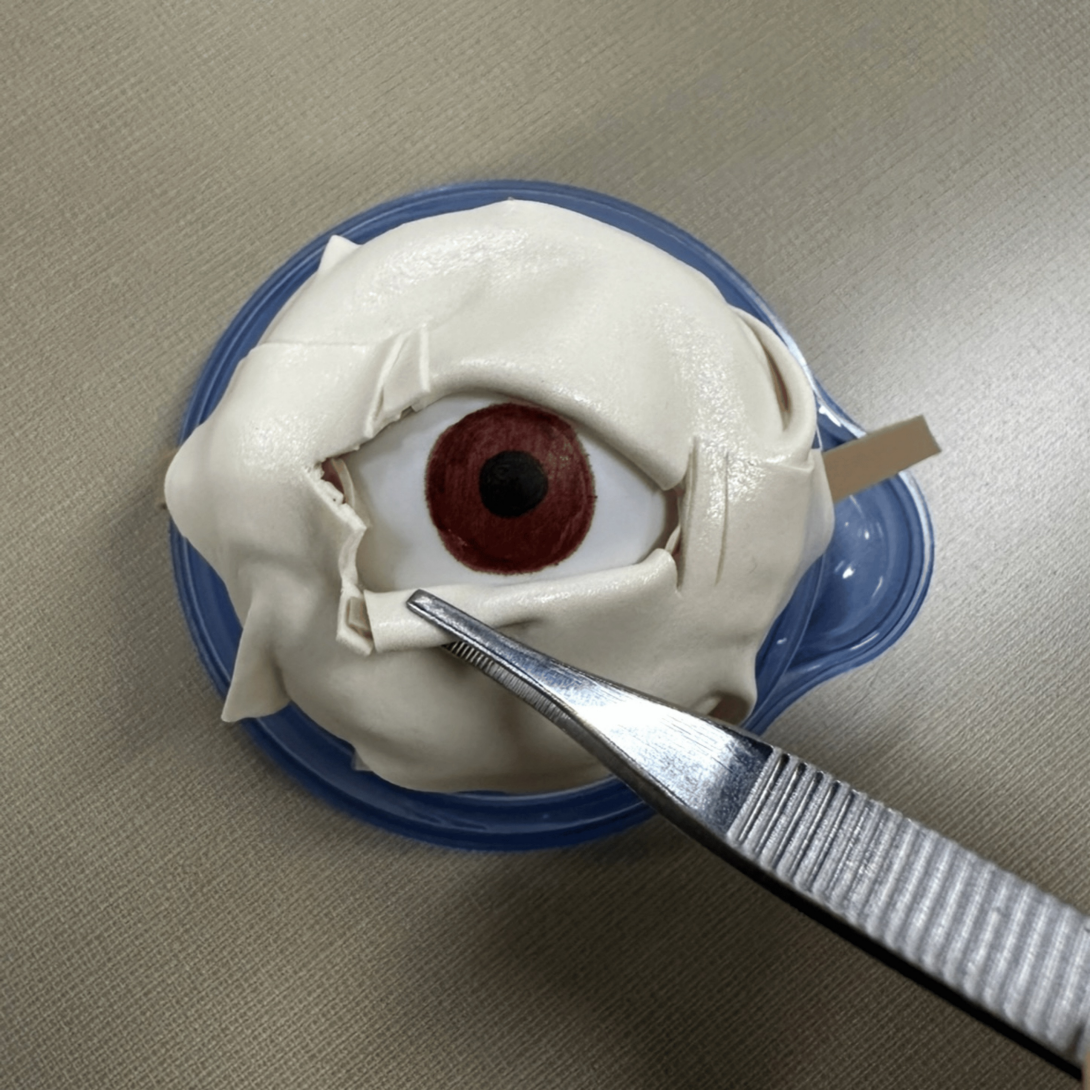
Lateral canthotomy trainer originally described by Kong and colleagues.

**Figure 2:**
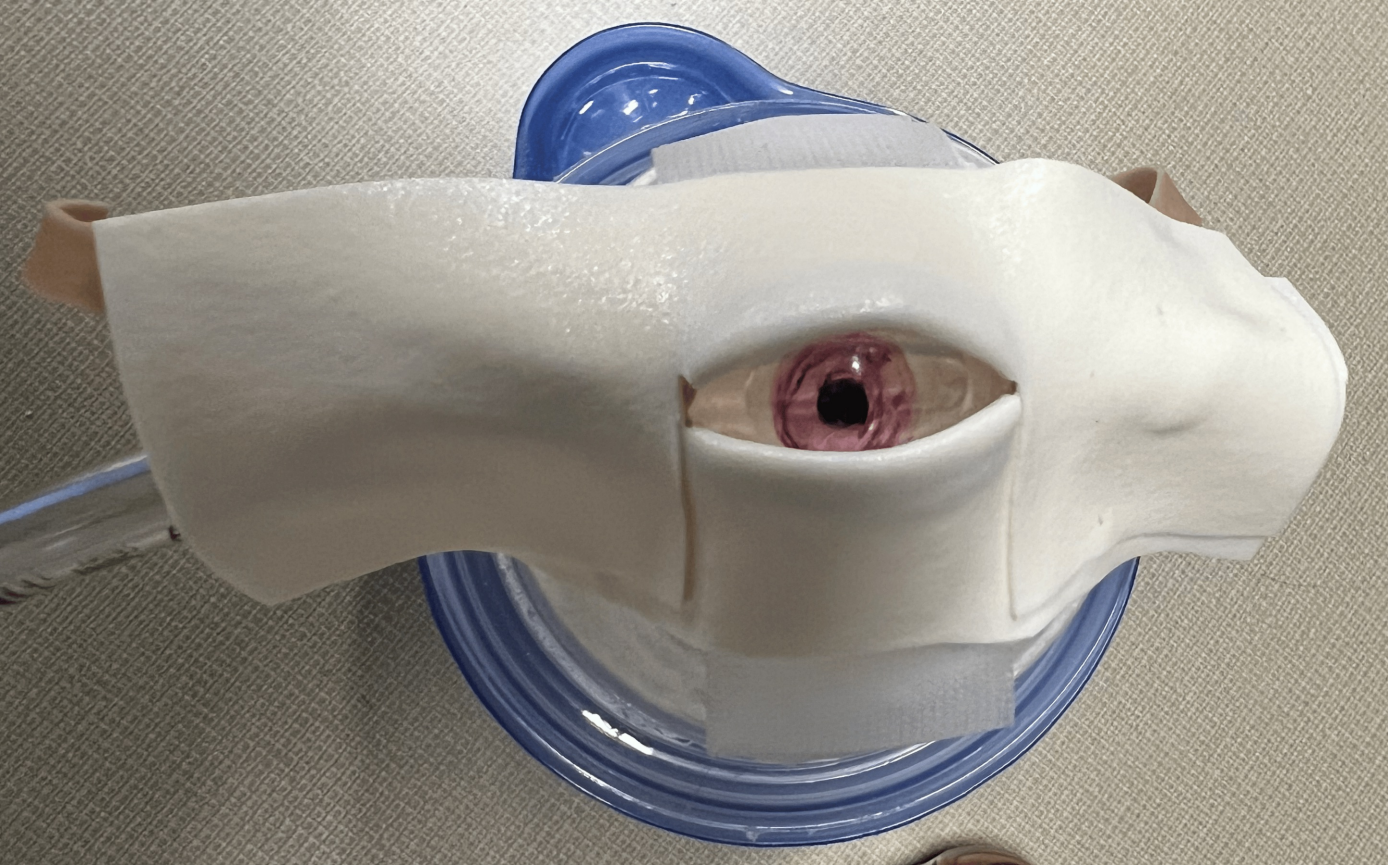
Modified lateral canthotomy trainer with foam tape "skin" layer and underlying canthus included. Here, we used an endotracheal tube cuff as the eye.

**Figure 3:**
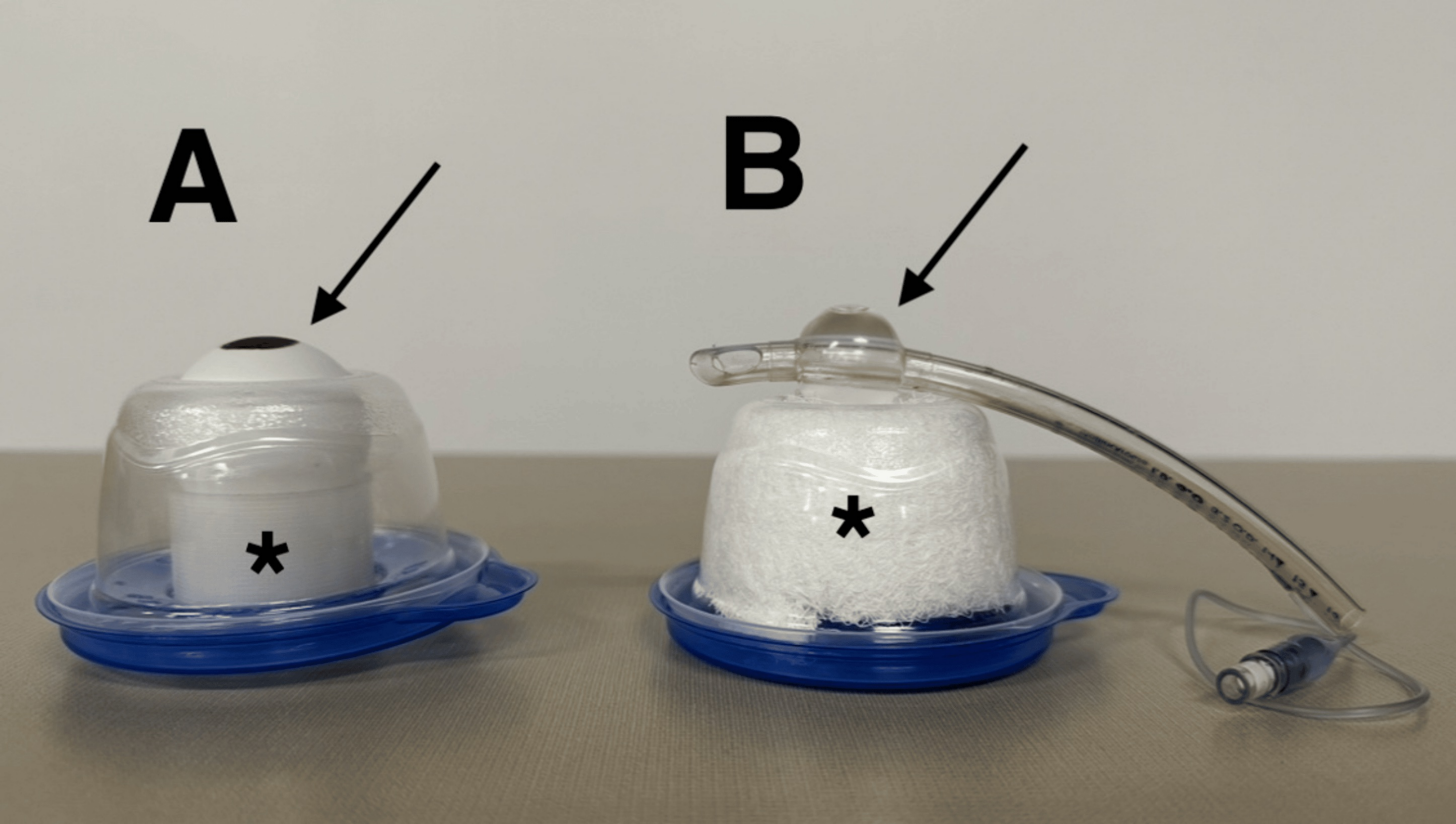
Side-by-side construction comparison of Kong’s trainer (A) and our modified version (B) with "globe" (arrow) and "hemorrhage" (*). The overlying replaceable foam tape “skin” layer with canthus is not pictured.

Kong’s original lateral canthotomy trainer used a plastic food storage container as the eye socket, a rubber band as the lateral canthus and crura, foam tape as the skin, and a table tennis ball as the globe (Figure 1). We experimented with several modifications to this design. To make the globe compressible, we first replaced the table tennis ball with a Foley catheter balloon. We discovered that the “low volume, high pressure” balloon generated pressures that far exceeded what would be expected in OCS. Ultimately, the cuff of a 6.0 ETT filled with normal saline worked best to simulate the globe (Figure 2). We experimented with a Cuffill digital manometer syringe (Medline, Northfield, Illinois) to set a precise cuff pressure. However, because we planned to have learners use a Tono-Pen tonometer device (Medtronic, Minneapolis, Minnesota) to measure eye pressure, we found that it was easier and more cost-effective to fill the cuff until the Tono-Pen measured the desired pressure (15-20 mmHg). We were able to achieve measurements as high as the upper limit of the Tono-Pen XL's range, which is 80 mmHg. With regard to placement of the globe, we positioned the eye on the outside and rested it on a Kerlix gauze roll (Covidien, Dublin, Ohio) placed inside the container. The gauze roll provided structural support, allowed for easier access to the pilot line in order to fill the cuff, and prevented the sharp edge of the plastic container’s circular cutout from rupturing the eye (Figure 3). Arranged in this way, the gauze roll is analogous to retro-orbital hemorrhage and can be increased or decreased in size. The rubber band “canthal tendon” presses the globe back against the “hemorrhage”, increasing its pressure. When filled with 8 mL of normal saline, the cuff’s diameter was 24 mm, which approximates that of a human adult eye [5]. We then used scissors to cut away unnecessary parts of the endotracheal tube.

**Figure 4:**
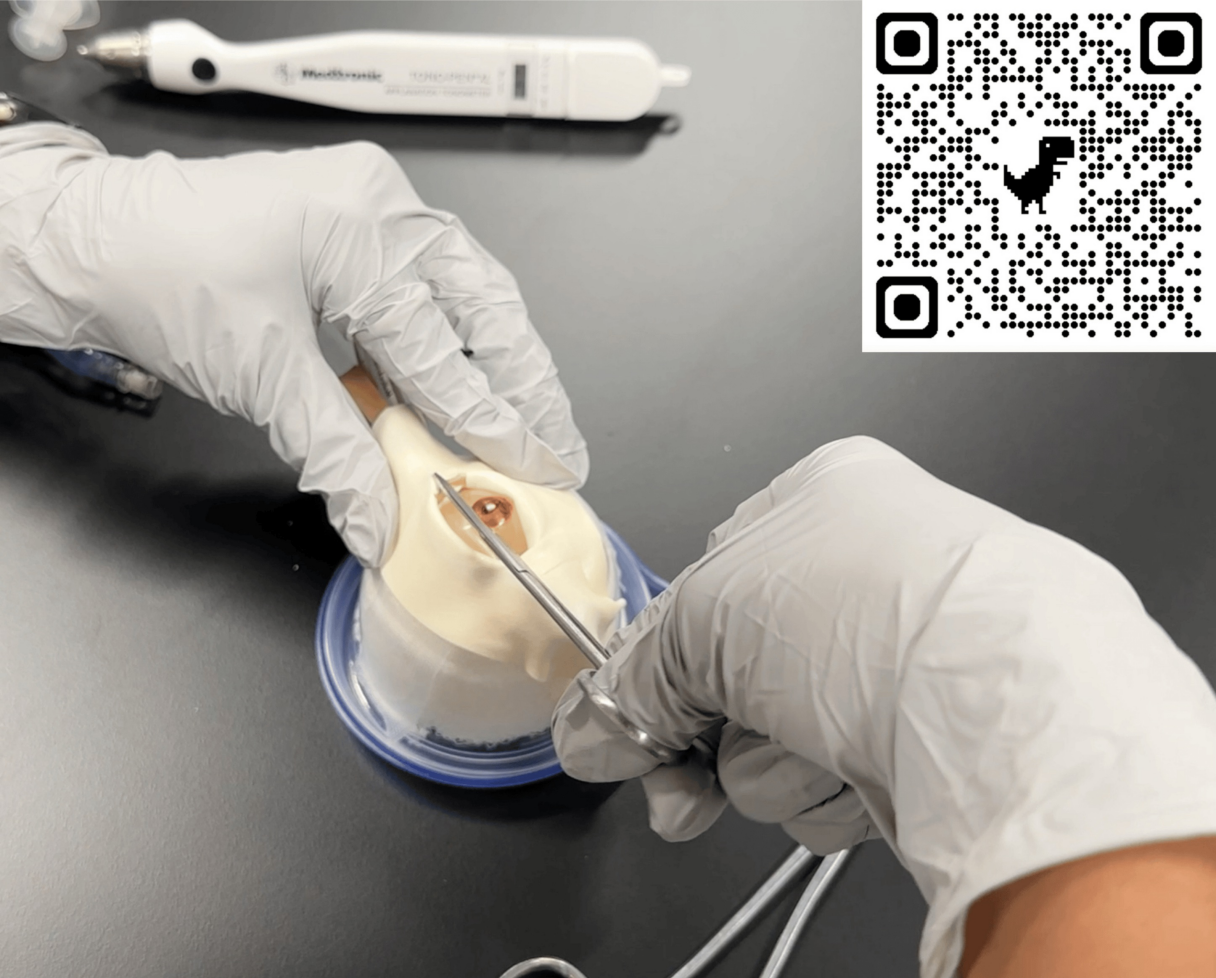
The modified trainer in use. A video demonstration can be viewed by scanning the QR code or following this link: https://youtu.be/ISvCWKa7ECU.

Internal model pressures could be measured using both Tono-Pen and iCare (iCare, Vantaa, Finland) devices. There are three methods to increase eye pressures: inject more saline into the cuff, apply the foam tape more tightly over the eye, or increase the amount of gauze packing behind the globe. With manual compression of the eye, there was a palpable difference between high and low pressures. Tactile differences allow learners to correlate palpation with measured IOP. We recommend injecting enough saline into the cuff to set the pressure to the desired level first (we used 15-20 mmHg), then applying the foam tape tightly enough over the eye so that critical orbital compartment pressures are reached. When the canthus and inferior crus are released, the pressure will return to the original amount, which signifies an effective procedure in the same way that it does for a live patient [6]. We found that when set up correctly, learners were able to measure IOP above 40 mmHg before the procedure and below 40 mmHg after canthal release. We recorded a demonstration of the trainer in use (Figure 4).

We used our trainer during scheduled emergency medicine residency simulation days with clinician learners of various levels of experience, from medical students to faculty. Sessions at our table lasted approximately 15-20 minutes each. Learners were asked to fill out both a pre- and post-activity survey. The survey asked the learner their current level of training and whether they had performed the procedure on a patient or a previous model. The survey then asked the following questions on a five-point Likert scale: “How comfortable are you taking an intraocular pressure?” and “How comfortable do you feel performing a lateral canthotomy?” Comfort levels measured were “extremely uncomfortable”, “somewhat uncomfortable”, “neither comfortable nor uncomfortable”, “somewhat comfortable”, and “extremely comfortable”.

After the initial survey was completed, we reviewed the use of the Tono-Pen and the procedure steps with participants, including a brief safety section. They were then asked to perform the procedures themselves, where we provided teaching during the procedure performance, as is usual for our teaching sessions. After the procedure, they completed the post-activity survey, which included the questions: “How comfortable are you taking an intraocular pressure after this training?” and “How comfortable do you feel performing a lateral canthotomy after this training?” We compared survey constructs in Stata version 12.2 (StataCorp, LLC, College Station, Texas) using a Wilcoxon matched-pairs test with a significance level of 0.05.

We had a total of 53 participants over the course of two simulation days. Of these participants, 23 were either medical students or first-year residents, 16 were second-year residents, 13 were third-year residents, and there was one fellow. Most participants (89%) had never performed a lateral canthotomy on a real patient, but 41% had experience with another simulation model. Each participant who completed a pre-survey also completed a post-survey. Comfort with performing tonometry improved after the session, with a median comfort level before the session of 4/5 (somewhat comfortable; IQR: 3-5) and a median comfort level after the session of 4.5/5 (somewhat comfortable/very comfortable; IQR: 4-5, P = 0.002). Comfort with performing lateral canthotomy and cantholysis improved after the session, with a median comfort level before the session of 2/5 (somewhat uncomfortable; IQR: 1-3) and a median comfort level after the session of 4/5 (somewhat comfortable; IQR: 3-4, P < 0.00001). Using a conservative Bonferroni correction for two comparisons (alpha/2), our findings would still be statistically significant at a level of alpha = 0.025.

## Discussion

Our modified lateral canthotomy trainer improved learners' comfort with cantholysis and IOP measurement. Other educators have created lateral canthotomy trainers with similar results [7-13]. Some examples are cadaveric, high-fidelity silicone, and animal models [7-9,14]. These anatomically realistic but single-use models are costly and difficult to handle or coordinate. Alternative cost-effective models have used materials such as 3D-printed spheres, foam gel, table tennis balls, or even hard-boiled eggs, each offering varying degrees of tactile realism [10-13,15]. Unlike our model, however, trainers built with these materials do not allow learners to measure IOP, which is an important confirmatory step in the procedure. Our trainer’s fluid-filled globe allows educators to incorporate tonometry measurements into teaching sessions. This capability enhances both procedural realism and skill transferability. Our trainer's globe is compressible and has a tactile and measurable pressure difference before and after canthal tendon release. It allows educators to set and adjust the desired IOP measurement. We did find that baseline comfort with tonometry was already high, likely because residents encounter tonometry much more frequently than lateral canthotomy. We discovered during the deployment of the trainer that the ETT can be ruptured if care is not taken. This simulates a globe rupture and requires learners to practice using caution around the globe while cutting.

Kong’s original model costs US$ 5 per trainer [4]. We used two trainers for our station and were able to get two uses of the “skin” for each trainer before switching them out. Our modification adds the cost of an ETT and a gauze roll. Both supplies are likely readily available to a medical educator. In the case that they are not, we were able to find an expired ETT online for US$ 5, and a gauze roll for the same price [16]. Fortunately, both of these additional items are reusable, keeping in mind that one should have extra ETTs available if a learner ruptures the globe. We recommend emphasizing that learners treat the cuff like a real globe in order to minimize ETT changes. Despite warning our participants, two additional ETT cuffs were ruptured and needed to be replaced in our session. Future studies could consider objective performance measures, include multiple institutions, and conduct long-term follow-up to see if skills are retained.

## Conclusions

We built a lateral canthotomy procedure trainer based on Kong’s simple model, substituting a fluid-filled ETT cuff for the table tennis ball. This modification enhances realism through tactile feel and incorporates measurable IOP assessment into procedural training. The fluid-filled globe allowed us to set and control IOP to be measured before and after cantholysis. It provided learners with the opportunity to manually compress the globe and feel the difference between a globe with normal and elevated pressure. Additionally, it provides a realistic opportunity to simulate accidental globe rupture, reinforcing cautious handling of instrumentation. The modified lateral canthotomy trainer demonstrated a statistically significant improvement in participants’ comfort with both procedures. We recommend our trainer's deployment in training programs with the following specialties: emergency medicine, ophthalmology, and trauma surgery. Future studies could evaluate the model’s impact on procedural accuracy and skill retention.
